# Annotation and sequence diversity of transposable elements in common bean (*Phaseolus vulgaris*)

**DOI:** 10.3389/fpls.2014.00339

**Published:** 2014-07-11

**Authors:** Dongying Gao, Brian Abernathy, Daniel Rohksar, Jeremy Schmutz, Scott A. Jackson

**Affiliations:** ^1^Center for Applied Genetic Technologies, University of GeorgiaAthens, GA, USA; ^2^US Department of Energy Joint Genome InstituteWalnut Creek, CA, USA; ^3^HudsonAlpha Institute of BiotechnologyHuntsville, AL, USA

**Keywords:** transposon, common bean, transposon database, evolution, ORF2

## Abstract

Common bean (*Phaseolus vulgaris*) is an important legume crop grown and consumed worldwide. With the availability of the common bean genome sequence, the next challenge is to annotate the genome and characterize functional DNA elements. Transposable elements (TEs) are the most abundant component of plant genomes and can dramatically affect genome evolution and genetic variation. Thus, it is pivotal to identify TEs in the common bean genome. In this study, we performed a genome-wide transposon annotation in common bean using a combination of homology and sequence structure-based methods. We developed a 2.12-Mb transposon database which includes 791 representative transposon sequences and is available upon request or from www.phytozome.org. Of note, nearly all transposons in the database are previously unrecognized TEs. More than 5,000 transposon-related expressed sequence tags (ESTs) were detected which indicates that some transposons may be transcriptionally active. Two Ty1-copia retrotransposon families were found to encode the envelope-like protein which has rarely been identified in plant genomes. Also, we identified an extra open reading frame (ORF) termed ORF2 from 15 Ty3-gypsy families that was located between the ORF encoding the retrotransposase and the 3′LTR. The ORF2 was in opposite transcriptional orientation to retrotransposase. Sequence homology searches and phylogenetic analysis suggested that the ORF2 may have an ancient origin, but its function is not clear. These transposon data provide a useful resource for understanding the genome organization and evolution and may be used to identify active TEs for developing transposon-tagging system in common bean and other related genomes.

## INTRODUCTION

Large portions of all sequenced plant genomes consist of highly repetitive sequences, such as transposable elements (TEs) and tandem repeats, which play crucial roles in plant genome organization. In contrast to other repetitive sequences, TEs are mobile genetic elements that can move within genome or via horizontal transfer between genomes (Roulin et al., 2008). In some cases, TE insertions can lead to deleterious or lethal effects to the organism in which they reside. TEs can impact genome structure and evolution. For example, centromeric retrotransposons (CRs) may be involved in the formation of functional centromeres (Jin et al., 2004). TEs serve as important components of heterochromatin maintaining chromosome stability and heterochromatic silencing (Grewal and Jia, 2007). TEs provide raw material for evolutionary novelties, such as new gene functions, expression patterns, and networks (Cordaux and Batzer, 2009). In addition, TEs have been used as mutagens to isolate genes and characterize biological functions.

Transposable elements are divided into two classes based on their transposition mechanisms: class 1 retrotransposons and class 2 DNA transposons. Class 1 incudes long terminal repeats (LTR) retrotransposons and non-LTR retrotransposons including long interspersed elements (LINEs) and short interspersed elements (SINEs). Class 2 contains the elements with terminal inverted repeats (TIRs) and internal sequences that encode enzymes necessary for movements (Wicker et al., 2007). Except for Helitron transposons (Class 2), transposition of all TEs generate target site duplications (TSDs) that range in size from 2-bp to more than 20-bp, according to TE superfamilies.

Transposable elements contribute significant fractions of many eukaryotic genomes and can dramatically impact genome structure, thus annotating TEs is a necessary activity (Juretic et al., 2004). Software tools have been developed to aid in the discovery of TEs based on different annotation strategies. RepeatMasker^1^ is widely used to annotate TEs, however, it depends on a user-defined TE library, the completeness of which affects annotation accuracy and efficiency. RECON was developed for *de novo* identification of TEs with multiple copies in genome (Bao and Eddy, 2002), thus low-copy TEs may be missed. TEs have structural features, including LTRs, TIRs, and TSDs, that can be used to distinguish them from genes and other genomic features. Several tools have been developed using structural features including LTR_STRUC (McCarthy and McDonald, 2003), LTR_FINDER (Xu and Wang, 2007), MITEs-Hunter (Han and Wessler, 2010) and others (Bergman and Quesneville, 2007). These programs predict intact TEs and truncated TEs may be missed. Another method to find TEs is based on homology. Autonomous TEs encode transposases which are relatively conserved within a transposon superfamily (Wicker et al., 2007), thus the conserved domains of transposases from different superfamilies can be used to search the genome and identify related TEs.

Common bean (*Phaseolus vulgaris L.*, 2*n* = 2*x* = 22) is an important food crop grown and consumed throughout the world and can be a major source of income for small farmers in some parts of the world (Blair et al., 2011). Common bean provides protein, fiber, micronutrients, and other valuable components that are absent or in low amounts in other crops and has been referred to as the “healthy people’s meat.” Common bean belongs to the legume family which is most notable for its ability to fix atmospheric nitrogen via symbioses with soil-borne rhizobia thereby reducing fertilizer costs for farmers. As a close relative of soybean (*Glycine max*, 2*n* = 2*x* = 40), common bean has been used to study domestication, effects of polyploidy and genome evolution of legumes (Lin et al., 2010; McClean et al., 2010; Bitocchi et al., 2012).

Genome sequences for soybean and pigeon pea (*Cajanus cajan*) have been reported (Schmutz et al., 2010; Varshney et al., 2011). As an important crop and a relative of these two sequenced genomes, the 521.1-Mbp common bean genome has been sequenced (Schmutz et al., 2014). In this study, we conducted genome-wide transposon annotation and developed a common bean transposon database. More than 5,000 transposon-related ESTs were found and suggesting that some TEs in common bean are transcriptionally active. We analyzed the sequence structures of LTR retrotransposons and identified 17 families that contain either the envelope-like protein or an additional open reading frame (ORF2) encoding an unknown protein. Our transposon database provides a valuable resource for the common bean genome annotation and comparative genomics within legumes, and may be used to discover active TEs for transposon-tagging in common bean.

## RESULTS

### CONSTRUCTION OF A TRANSPOSON DATABSASE FOR COMMON BEAN

Several approaches were employed to discover transposon sequences in the common bean genome. First, sequences for 15 BACs (2.2 Mb) were obtained from GenBank and screened with LTR_FINDER to develop a control set of transposons. In addition, all against all BLASTN searches were performed for the 15 BAC sequences. A total of 12 LTR retrotransposon families were identified from these BAC sequences. To identify additional LTR retrotransposon in common bean, LTR_FINDER (Xu and Wang, 2007) was used to screen the draft genome sequence of common bean (V1.0 available from www.phytozome.net) that yielded 10,349 potential LTR retroelements. The boundaries, TSDs, structures of all these sequences were manually inspected which resulted in 2,288 sequences being designed as LTR retrotransposons and another 8,061 sequences were discarded as they were either tandem repeats, incomplete transposons or other sequences. The 2,288 elements were classified into 165 distinct families according published criteria (Wicker et al., 2007), this includes the 12 families identified in the 15 BACs and the two previously described LTR retroelements, Tpv2-6 (Garber et al., 1999) and pva1-118d24-re-5 (Wawrzynski et al., 2008). To classify these LTR retroelement families, representative elements for each of 165 families were used to conduct BLASTX or/and BLASTP searches. Sixty-five and 78 families were classified into Ty1-copia and Ty3-gypsy group, respectively. We were not able to group the other 22 families as these elements either encoded no retrotransposase or the encoded proteins were very short. Numerous LTR retroelements were found with exactly identical LTRs which indicates recent insertions of these retroelements and that some of them may still be active in common bean.

Long interspersed elementss are ubiquitous components of many characterized organisms. For instance, they are present in more than 500,000 copies in human comprising ∼17% of the genome (Cordaux and Batzer, 2009). LINEs are also abundant in plants and play important role in plant genome evolution (Feschotte et al., 2002). Typical LINEs contain a Poly (A) tail and a reverse transcriptase (RT) that shares little similarity to those of LTR retrotransposons. Additionally, complete LINEs usually flanked by TSDs that vary in length from 5-bp to more than 20-bp (Kojima, 2010). These features can be used to discover the LINEs in common bean. We used the conserved domain of the RT of LINEs (Feschotte et al., 2002) to conduct TBLASTN against the whole genome sequence and the BAC sequences. All hits (*E* value < 10^-20^) and flanking sequences (10-kb for each side) were extracted and used to search the genome and GenBank to identify the poly (A) motif and TSDs. We were able to identify several complete LINEs. For instance, a 12,917-bp LINE named pvLINE1 encodes an endonuclease and retrovirus RT and contains a 35-bp poly (A/T) tail. pvLINE1 was flanked by 29-bp TSD. However, not all LINEs can be defined as complete elements as they may have accumulated mutations and/or internal rearrangements or due to frequent premature arrest of reverse transcription which can result in truncated LINEs at the 5′ end (Brouha et al., 2003; Cordaux and Batzer, 2009).

A total of 12 superfamilies of DNA transposons have been identified across kingdoms of which six superfamilies are found in plants. These include Mutator-like element (MULE), hAT, CACTA, PIF/Harbinger, Helitron, and Tc1/Mariner; elements such as P elements and PiggyBac are present in animals only or other organisms (Feschotte and Pritham, 2007; Wicker et al., 2007). To detect DNA transposons in common bean, the conserved domains of the six superfamilies were used as queries to conduct TBLASTN searches. Hits were found for MULE, hAT, CACTA, PIF/Harbinger, and Helitron transposons; however, no hit was found for the Tc1/Mariner superfamilies. We then used MITEs-Hunter (Han and Wessler, 2010) to find DNA transposons without transposases, the output sequences were manually inspected and their classifications were determined according to the terminal repeats and TSDs. For example, CACTA elements were defined by the “CACTA…ATGTG” terminal motifs and 2–3 bp TSD. We identified 348 CACTA sequences, 45 MULEs, 23 hATs, and 39 Helitrons. An additional 46 repetitive sequences could not be grouped because they encoded no protein and did not have characteristic terminal motifs. All identified transposons were combined into a common bean transposon library that includes 791 representative transposon sequences (**Table 1**) and is 2.12 Mb in size.

**Table 1:** Summary of the common bean transposon database.

Transposon superfamily	Number of transposon in the database
Class 1	285
LTR retrotransposons	176
Ty1-copia	73
Ty3-gypsy	80
Unclassified	23
Non-LTR retrotransposon	109
LINE	105
SINE	4
Class 2	460
CACTA	348
hAT	23
MULE	45
Helitron	39
PIF/Harbinger	5
Unclassified	46
Total	791

### TRANSPOSON-RELATED EXPRESSED SEQUENCE TAGS (ESTs)

**FIGURE 1:**
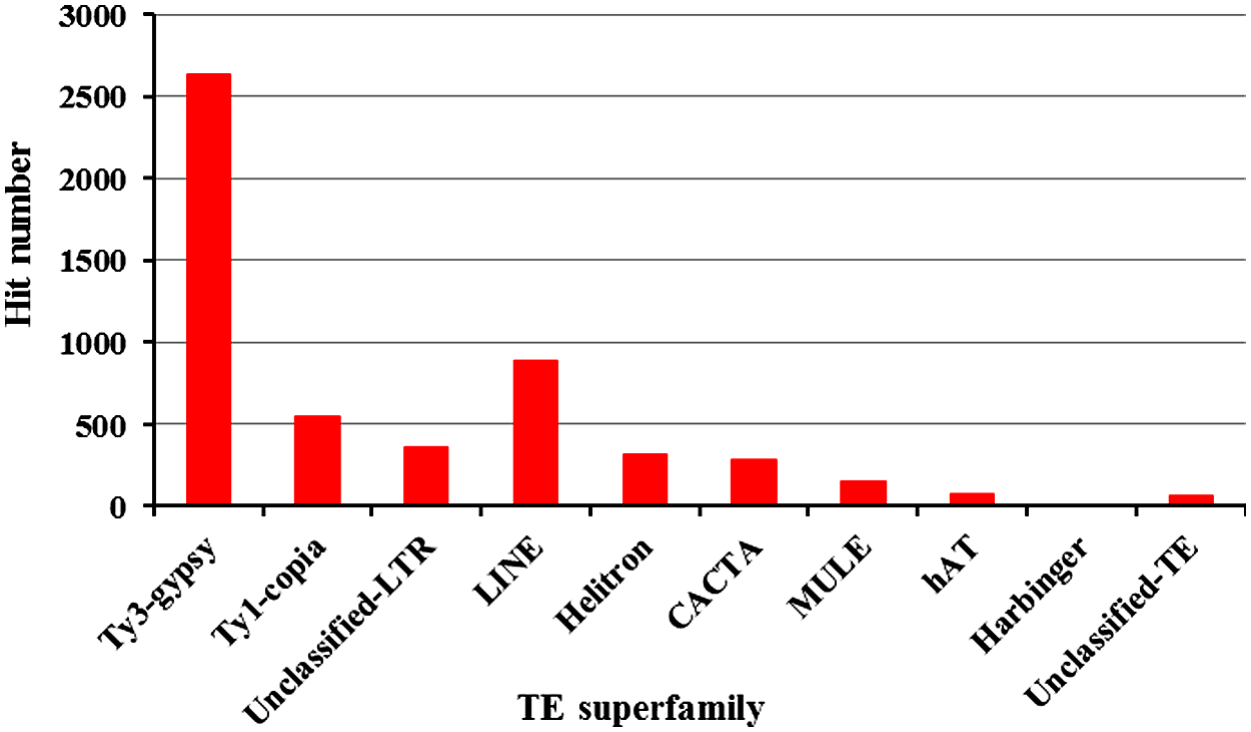
**Summary of common bean-derived ESTs that share sequence similarity with different transposons**.

A total of 148,267 ESTs from common bean were deposited in GenBank as of July 1, 2012. We hypothesized that some TEs may be transcriptionally active and that transposon-related ESTs may be detected. To test this, all ESTs were used to search against our common bean transposon database. A total of 5,328 ESTs were found to have significant sequence similarity to TEs (*E* value < 10^-5^). Excepting SINEs, each TE had EST hits though the numbers vary among the various TE superfamilies (**Figure 1**). We found 2,633 Ty-gypsy-related ESTs that account for 49.4% of the expressed TE sequences. A total of 546 (10.2%) and 356 (6.7%) ESTs were found to have significant similarity to Ty1-copia and unclassified LTRs, respectively. LINE elements are frequently transcribed and represent the most abundant TEs in mammals (Cordaux and Batzer, 2009). However, active LINEs are not common in plant genomes. It is interesting that we detected 893 (16.8%) LINE-related ESTs which indicate that some LINEs are highly expressed in common bean. Furthermore, a total of 838 ESTs related to DNA transposons were found, including 322 Helitrons, 284 CACTAs, 156 MULEs, 72 hATs, and 4 PIF/Harbingers, which cumulatively represent ∼15.7% of the TE-related ESTs.

### STRUCTURAL ANALYSIS OF LTR RETROTRANSPOSONS

**FIGURE 2:**
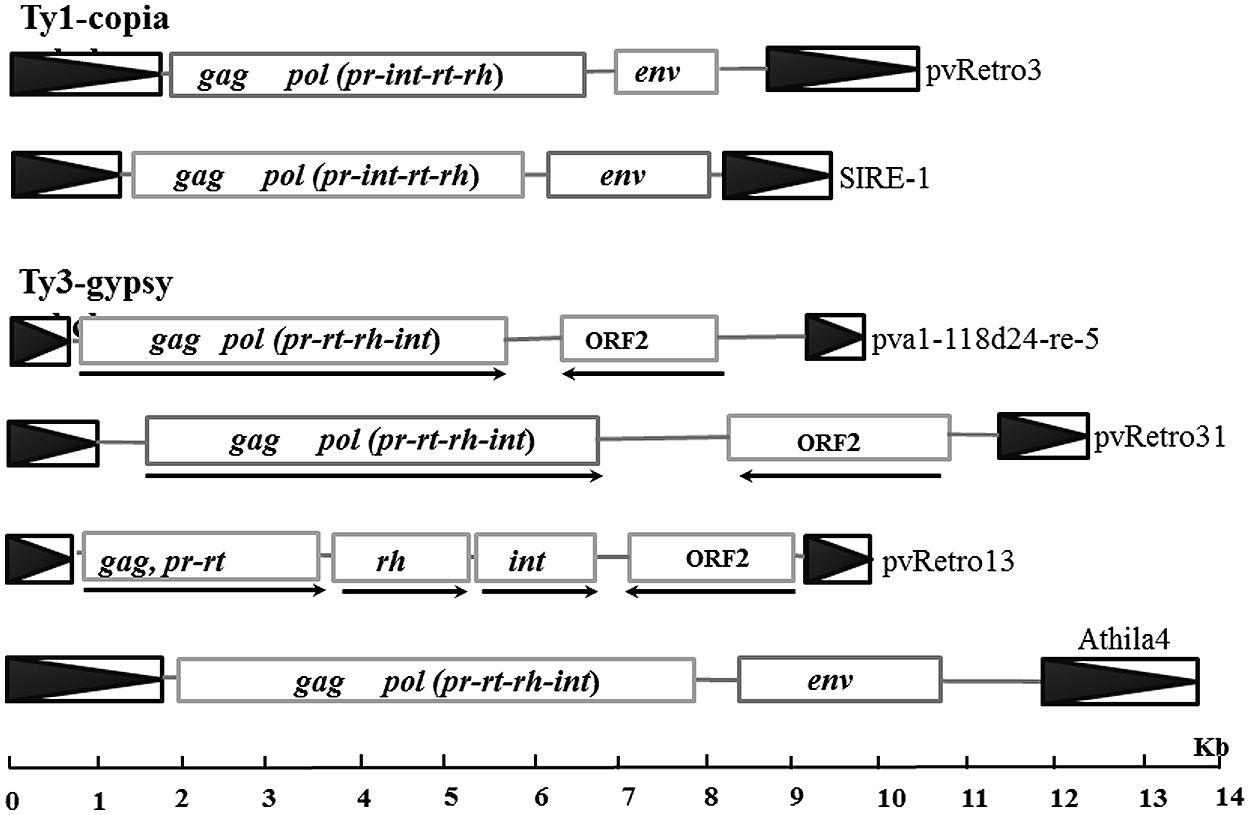
**Sequence structures of different LTR retrotransposons.** Boxes with black triangles represent transposon LTRs. Rectangles indicated open reading frames (ORF) encoding retrotransposases or other proteins and arrows indicate the transcription orientations for retroelements with ORF2.

Previous studies have shown that some plant LTR retrotransposons contain an extra ORF encoding envelope-like proteins, including two Ty1-copia elements, SIRE1 in soybean (Laten et al., 1998) and ToRTL1 in tomato (Tam et al., 2007), and three Ty3-gypsy elements, Cyclops2 in pea (Chavanne et al., 1998), Athila4 of Arabidopsis and Calypso of soybean (Wright and Voytas, 2002). To determine whether LTR retroelements of common bean carry an extra ORF coding envelope-like proteins, the internal regions of 65 Ty1-copia and 78 Ty3-gypsy families were annotated using FGENESH^2^ and GENSCAN^3^. 96.9% (63/65) of Ty1-copia and 80.8% (63/78) of Ty3-gypsy transposon families contain only retrotransposase encoding ORFs. Interestingly, two Ty1-copia families and 15 Ty-gypsy families were found to have an extra ORF located between the retrotransposase ORF and 3′ LTR (**Table 2**; **Figure 2**). The extra ORFs do not share similarity with any retrotransposase sequences.

**Table 2:** Common bean LTR retrotransposons with the envelope-like protein or an additional open reading frame (ORF2).

TE name	Subclass	TE size (bp)	LTR size (bp)	Retrotransposase (aa)	Env/ORF2 size (aa)
pvRetro3	Ty1-copia	10,278	1,773	1,541	381
pvRetro4	Ty1-copia	10,443	1,706	1,267	393
pva1118d24-re-5	Ty3-gypsy	9,351	457	1,648	568
pvRetro9	Ty3-gypsy	9,062	456	1,321	436
pvRetro13	Ty3-gypsy	9,899	579	923,300,203	631
pvRetro26	Ty3-gypsy	13,601	958	1,523	849
pvRetro31	Ty3-gypsy	12,141	953	1,581	688
pvRetro36	Ty3-gypsy	11,784	1,177	1,322	508
pvRetro37	Ty3-gypsy	11,540	845	1,528	437
pvRetro38	Ty3-gypsy	9,640	559	1,278	524
pvRetro48	Ty3-gypsy	12,722	1,158	1,302	939
pvRetro51	Ty3-gypsy	11,975	1,128	1,360	554
pvRetro52	Ty3-gypsy	11,212	844	847	424
pvRetro65	Ty3-gypsy	10,638	462	1,250	211
pvRetro124	Ty3-gypsy	9,194	492	1,113	402
pvRetro137	Ty3-gypsy	8,680	989	229	535
pvRetro143	Ty3-gypsy	8,796	1,239	275	347

*The proteins that are necessary for retrotransposition of pvRetro13 were encoded by three ORFs (**Figure 2**), but the internal sequence for the other 14 families contained only one ORF encoding the retrotransposase.*

Two Ty1-copia families with the extra ORF, pvRetro3 and pvRetro4, shared less than 30% similarity between their LTRs but their internal regions had 76% similarity over 5 kb indicating that these two elements likely diverged from a common ancestral LTR retroelement. To define the potential function of the extra ORFs, the 381-aa protein of pvRetro3 and 393-aa protein of pvRetro4 were used to search against GenBank and the Gypsy database^4^, however, no conserved domain was found for either of these two proteins. pvRetro3 and pvRetro4 were then used to perform BLASTN searches, the internal sequences from both elements share more than 65% sequence similarity over 2 kb with that from MtRetro3 (AC144724:31185-43634) from *Medicago truncatula*; LjRetro3 (AP006091:15667-26573) in *Lotus japonicas* and Grm6 (gmp1-12a14-re-4, FJ198003) in soybean. It is interesting that MtRetro3 and LjRetro3 also contain two ORFs, the retrotransposase-encoded ORF (1597 and 1584-aa for MtRetro3 and LjRetro3, respectively) and an extra ORF (774 and 793-aa). However, Grm6 had only one ORF encoding a 1768-aa retrotransposase. Notably, the 774-aa protein of MtRetro3 shows sequence similarity to the envelope-like proteins of SIRE1 (*E* value = 6e × 10^-4^) and TorTL1 (*E* value = 2e × 10^-10^). It also exhibits similarity to pvRetro3 (1e × 10^-15^), pvRetro4 (7e × 10^-16^), and LjRetro3 (4e × 10^-16^). These results suggests that the pvRetro3 381-aa and pvRetro4 393-aa proteins were likely envelope-like proteins but have either diverged or been truncated.

We also found 15 Ty3-gypsy retrotransposon families that contained an extra ORF (ORF2) in addition to the ORF(s) that encode the retrotransposase (complete or partial; **Figure 2**; **Table 2**). Unlike the two Ty1-copia families and other retroelements with envelope-like proteins, the retrotransposase and the ORF2-encoded protein of all these 15 retroelements were in opposite transcriptional orientations. Furthermore, no detectable sequence similarity was found between described envelope proteins and the proteins encoded by the ORF2s. However, the ORF2-encoded proteins shared sequence similarity with each other suggesting a common origin. For instance, the protein of pva1118d24-re-5 shows significant similarity to that of pvRetro13 (1e × 10^-66^) and pvRetro124 (2e × 10^-39^). In addition, some of the proteins contain a conserved domain, structural maintenance of chromosomes (SMC), that binds DNA and acts in organizing and segregating chromosomes for partitioning (Marchler-Bauer et al., 2011).

Open reading frame 2-encoded proteins were then used conduct TBLASTN searches to detect if homologous sequences were present in other genomes. Thirteen, 40, and 226 significant hits (*E* value < 1 × 10^-5^) were found in* G. max, M. truncatula* and *L. japonicas*, respectively. All hits and their flanking sequences were manually inspected and a complete elements, gtd1-136a20-re-1 (FJ402912) from *G. max* and LjRetro26 (AP009775:48573-58015) from *L. japonicas*, were identified. No intact element was identified in *M. truncatula* suggesting complete element may have been degraded, missed by our investigation or was not captured in the sequence assembly. gtd1-136a20-re-1 and LjRetro26 have ORF2s encoding 868 and 461-aa protein, respectively. The two retrotransposons likely are autonomous elements as they encode >1800-aa retrotransposase containing all domains necessary for retrotransposition.

### PHYLOGENETIC ANALYSIS OF LTR RETROTRANSPOSONS

**FIGURE 3:**
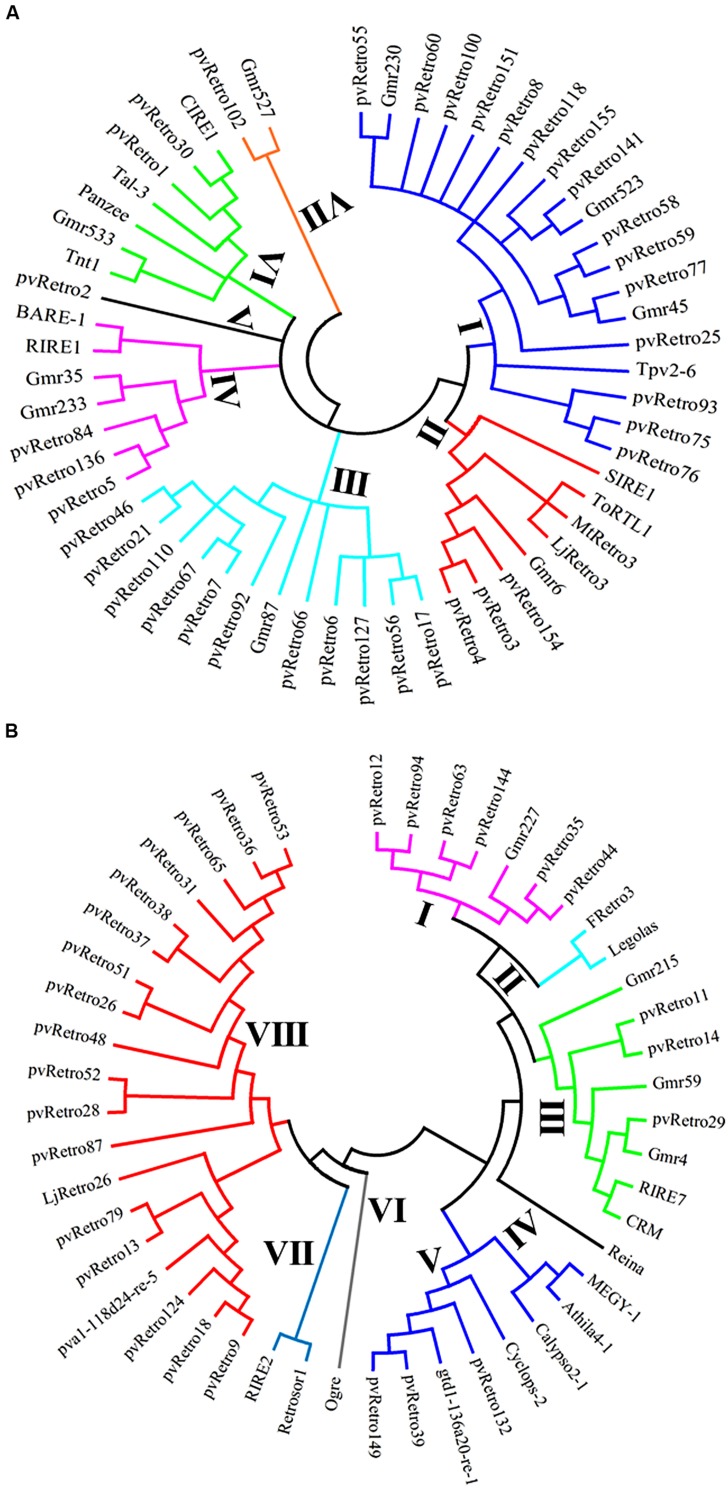
**Phylogenetic tree using the conserved RT domains of Ty1-copia **(A)** and Ty3-gypsy **(B)** LTR retrotransposons**.

To gain insight into evolutionary relationships between LTR retrotransposons from common bean and other plants, two phylogenetic trees were constructed based on conserved RT domains of LTR retrotransposons. Among 65 Ty1-copia families in common bean, 37 families carry nearly complete RT domains, whereas the other 28 families contain either short or no RT domains. The RT sequences of 56 Ty1-copia elements, including 37 families from common bean, 10 families from soybean, and 9 elements from other plants were used to build a phylogenetic tree. These retrotransposons were clustered into seven clades, clade I contains 19 elements whereas clade V only has pvRetro2 (**Figure 3A**). Two retroelements that encode envelope-like proteins, SIRE1 in soybean and TorTL1 in tomato, were grouped into clade II together with pvRetro3, pvRetro4, MtRetro3, and LjRetro3. Even though pvRetro3, pvRetro4, and SIRE1 fell into a same clade, pvRetro3, and pvRetro4 likely shared more recent ancestry with Grm6 from soybean than with SIRE1 as both pvRetro3 and pvRetro4 show higher sequence similarity with Grm6 than SIRE1 at the nucleotide level.

**FIGURE 4:**
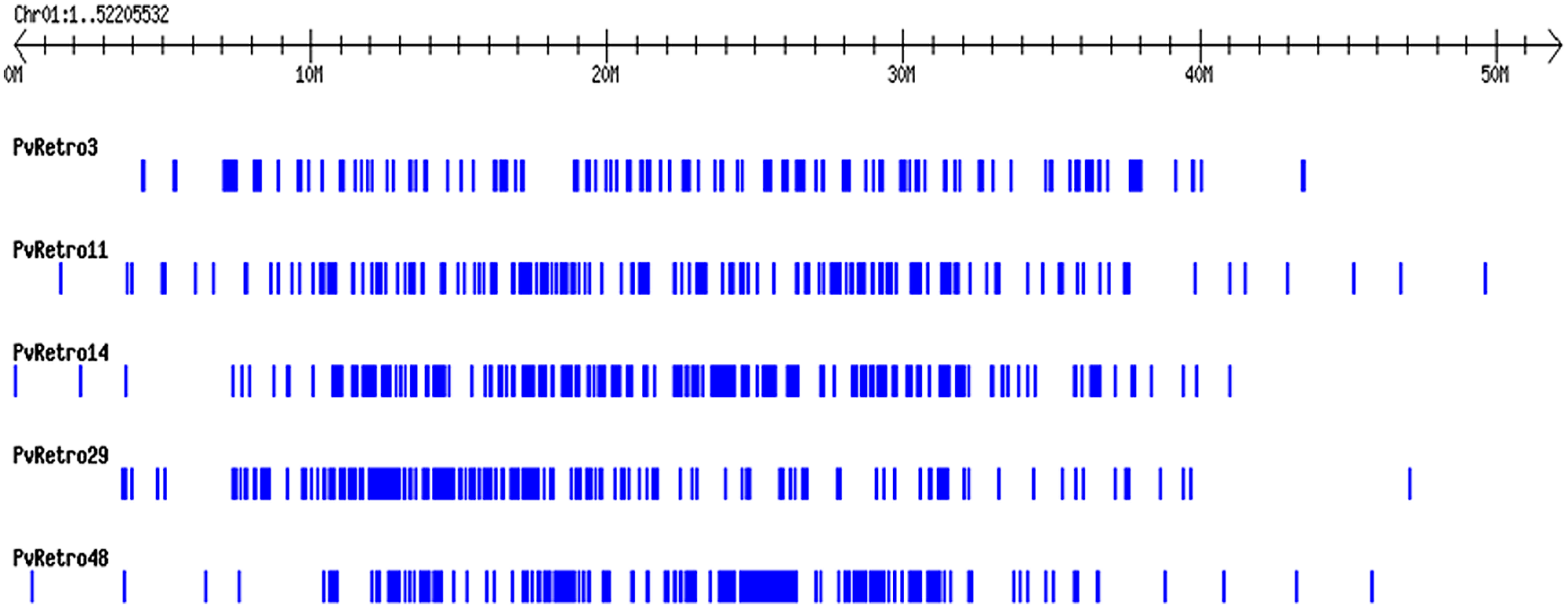
**Distribution of five LTR retroelement families on the chromosome 1 of common bean.** The distribution patterns of the five transposons across other nine chromosomes are not shown as they are similar to chromosome 1.

Another phylogenetic tree was made using RT domains of 48 Ty3-gypsy families including 30 common bean transposons and 5 soybean retroelements. The Ty3-gypsy retrotransposons were grouped into eight clades (**Figure 3B**). Clades IV and VI contain only one element, whereas, clade VIII contains 19 retroelements. It is worth to note that all 15 families containing ORF2 (**Table 2**) but pvRetro137 and pvRetro143 that do not encode RT domains were clustered into clade VIII. Three LTR retrotransposons encoding envelope-like proteins, Cyclops2 in pea (Chavanne et al., 1998), Athila4 of Arabidopsis and Calypso in soybean (Wright and Voytas, 2002) fell into clade V. Interestingly, three elements, pvRetro11, pvRetro14, and pvRetro29 were classified together the centromeric retrotransposons, CRM in maize (Jin et al., 2004) and RIRE7 in rice (Kumekawa et al., 2001), and three elements, Gmr4, Gmr59, and Gmr215, which may be centromeric retroelements in soybean (Du et al., 2010a). pvRetro11, pvRetro14, and pvRetro29 were used to search against the soybean transposon database^5^ (Du et al., 2010b). The internal sequences of all three retroelements show ∼80% sequence similarity with Gmr4 over a 300-bp region but the LTRs from pvRetro11, pvRetro14 and pvRetro29 share no detectable similarity with Gmr4 or other any soybean transposon. To determine if pvRetro11, pvRetro14, and pvRetro29 are located in centromeric regions in common bean, the genomic distributions of three retrotransposons were investigated. Although each family varies in distribution, pvRetro11, pvRetro14, and pvRetro29 were dispersed across the common bean genome (**Figure 4**, chromosome 1 shown as illustrative of the other 10 chromosomes).

## DISCUSSION

TEs are ubiquitous component in all eukaryotic genomes complicating assembly of whole genome shotgun sequencing projects and annotation of reference genomes. Although numerous computational tools have been developed that have accelerated the process of identifying transposons (Bergman and Quesneville, 2007), it still is a time-consuming and challenging task to annotate these abundant and often highly divergent elements. Using a single annotation approach, either homology- or structure-based, will render a biased transposon annotation. In this study, we annotated TEs in common bean by a combination of homology and structure approaches and developed a 2.12-Mb transposon database of common bean which includes 791 transposon reference sequences. Heretofore, only a few transposons of common bean had been described (Garber et al., 1999; Wawrzynski et al., 2008), thus the overwhelming majority of transposons in our database represent previously unrecognized TEs in common bean. We should note that we do not include all members for each transposon family in the transposon database as it will enlarge the size of TE library and impede computational analyses.

Transposon databases are useful resources for both applied and basic research in plants (Ouyang and Buell, 2004; Du et al., 2010b). First, TEs are one of the most conspicuous genomic features in plants and have been shaping host genomes through insertions, deletions, and illegitimate recombination. TEs also provide raw material for the evolution of new genes and regulatory networks (Feschotte and Pritham, 2007; Cordaux and Batzer, 2009). Therefore, the transposon database is useful not only for genome annotation but also for the study of gene/genome evolution, domestication and population genetics in plants (Panaud, 2009). Second, a transposon library can aid in comparative analyses. Syntentic relationships between common bean and other legumes have been established (Lin et al., 2010; McClean et al., 2010; Bitocchi et al., 2012); however, it is still not understood what role structural rearrangements may have played in legume genome divergence and speciation. The availability of the genome sequences from soybean (Schmutz et al., 2010), pigeon pea (Varshney et al., 2011) and other legumes will allow us to compare the transposon organization and dynamics at genome level and to define the evolutionary events mediated by transposons. Third, the common bean transposons may be helpful for annotating other related genomes. Numerous transposons were identified in this study that are not restricted to the common bean as homologs can be found in soybean, *M. truncatula*, *L. japonicas,* and other genomes. Thus, the common bean transposon database offers a good resource to predict the transposons in closely related genomes. Finally, the transposon dataset may be used to identify active transposons in common bean. Active endogenous transposons can be used for insertional mutagenesis. For instance, the Ac/Ds elements and the Mutator transposons have been used for gene discovery in maize (Raizada et al., 2001; Vollbrecht et al., 2010). In this study, we identified 5,328 transposon-related ESTs. It may be that transposons have involved in gene construction and that these ESTs are genic fragments or there may be transcriptionally active transposons in common bean.

LTR retrotransposons containing envelope-like proteins are not common in plants. Legume genomes are an exception, however, as retroelements with envelope-like protein were identified in soybean (Laten et al., 1998; Wright and Voytas, 2002), pea (Chavanne et al., 1998) and *L. japonicas* (Holligan et al., 2006). In this study, we found two Ty1-copia families, pvRetro3 and pvRetro4, that contain an ORF encoding an envelope-like protein. We also identified retroelement MtRetro3 in *M. truncatula* and LjRetro3 in *L. japonicas* which both encode a protein showing sequence similarity to other envelope-like proteins. Phylogenetic analysis indicated that pvRetro3, pvRetro4, MtRetro3, and LjRetro3 were related to the SIRE1 element that encodes retroviral envelope-like protein in soybean (Laten et al., 1998). It is possible that the envelope-like proteins in common bean, and other legumes, may have a role in horizontal transfer of the LTR elements.

Of note were the 15 Ty3-gypsy families with an extra ORF (ORF2) encoding proteins in opposite transcriptional orientation to the retrotransposase (**Table 2**; **Figure 2**). In addition to retroelements with envelope-like proteins, the Retrosat2/FRetro3 family from rice also has an extra ORF, ORF0, located between 5′ LTR and retrotransposase that encodes the protein involved in metabolism (catabolic processes; Gao et al., 2009, 2012). The ORF2-encoded proteins do not share any sequence similarity with either envelope-like protein or the ORF0-protein of Retrosat2 and phylogenetic analysis indicated that 15 retrofamilies with ORF2 were grouped into different clades from elements with the envelope-like protein (**Figure 3B**). These results indicate that the ORF2-encoded protein is likely not related to envelope-like proteins. The conserved SMC domain (Marchler-Bauer et al., 2011) was found in some ORF2-encoded proteins and may have a functional role in the biology of these transposons though that remains ambiguous. ORF2 proteins were also detected other legumes indicating an ancient origin before the divergence of the legumes.

## MATERIALS AND METHODS

### DATA SOURCES

The common bean genome sequence, including chloroplast and mitochondrion, was generated by the common bean genome sequencing project (available at www.phytozome.org; Schmutz et al., 2014). Fifteen common bean BAC sequences, DQ205649 (Abdelnoor et al., 2006), DQ323045 (Kami et al., 2006), FJ817289–FJ817291 (David et al., 2009), and GU215957–GU215966 (Lin et al., 2010), and EST sequences of common bean were downloaded from GenBank.

### SEQUENCE ANALYSIS

We conducted transposon annotation using different approaches according to the sequence features of various transposon superfamilies. For annotating LTR retrotransposons, the genome sequence was screened with LTR_FINDER (Xu and Wang, 2007) using default settings except that we used a 50 bp of minimum LTR length and 50 bp of minimum distance between LTRs. LINEs were predicted by the retrotransposase and polyA motif. The SINEs were detected based on the polyA structure feature and combined with BLASTN searches. To annotate DNA transposons, the conserved domain of each superfamily (Wicker et al., 2007) was used to conduct TBLASTN against the genome sequence and hit sequences (*E*-value < 1 × 10^-20^) and flanking sequences (10-kb for each side) were extracted and used for BLASTN searches to define transposon boundaries. In addition, we used the MITEs-Hunter program (Han and Wessler, 2010) to identify small DNA transposons that do not encode proteins and may be missed by the transposase searches. All annotated transposons were conducted all against all BLAST searches to group them into different families by following previous publication (Wicker et al., 2007) or to remove the highly similar trtansposons. To simplify the transposon database, only the representative elements were included which are intact elements or longer transposon fragments and share more than 70% sequence similarity with other elements from same family or group.

### SEQUENCE STRUCTURE AND PHYLOGENETIC ANALYSIS OF LTR RETROTRANSPOSONS

The sequence structures of LTR retrotransposons were predicted by the FGENESH^6^ and GENSCAN^7^ programs. The predicted proteins were used to search GenBank and the Gypsy database^8^ to identify functions and the RT conserved domains.

The phylogenetic trees were constructed based on the RT conserved domains of LTR retroelements from common bean, soybean, and other organisms. The alignments of multiple RT sequences were generated with CLUSTALW program^9^ with default options and the ambiguous regions were deleted. Phylogenetic trees were generated with MEGA4^10^ using the neighbor-joining method. A total of 22 retroelements from different legumes were use, including *Cyclops-2* (AJ000640) and* Ogre* (AY299398) from pea (*Pisum sativum* L.); Panzee (AJ000893) from Pigeon pea (*Cajanus cajan*); MEGY-1 (AC146683) from *M. truncatula* and 15 elements from soybean. In addition to SIRE1-4 (AY205608) and Calypso2-1 (AF186183), 13 LTR retroelements in soybean, including nine Ty1-copia elements (Gmr6, Gmr35, Gmr45, Gmr87, Gmr230, Gmr233, Gmr523, Gmr527, and Gmr533) and four Ty3-gypsy elements (Gmr4, Gmr5, Gmr215, and Gmr227), were obtained from the soybean transposon database^11^ (Du et al., 2010b). In addition, 14 retrotransposons from other plants also were selected which include Athila4-1(AC007209), Legolas (AC006570), and Ta1-3 (X13291) from *Arabidopsis thaliana*; RIRE2 (AB030283) and RIRE7 (AB033235) from rice (*Oryza sativa*); RIRE1 (D85597) from *O. australiensis*; FRetro3 (GU369679) from *O. brachyantha*; Reina (U69258), and CRM (AY129008) from maize (*Zea mays*); Retrosor1 (AF098806) from sorghum (*Sorghum bicolor*); BARE-1 (Z17327) from barley (*Hordeum vulgare*); Tnt1 (X13777) from tobacco (*Nicotiana tabacum*; CIRE1 (AM040263) from *Citrus sinensis* and ToRTL1 (U68072) from tomato (*Lycopersicon esculentum*).

## Conflict of Interest Statement

The authors declare that the research was conducted in the absence of any commercial or financial relationships that could be construed as a potential conflict of interest.

## Footnotes

1 http://www.repeatmasker.org

2 http://linux1.softberry.com

3 http://genes.mit.edu/GENSCAN.html

4 http://gydb.org/index.php/Main_Page

5 http://soybase.org/soytedb

6 http://linux1.softberry.com

7 http://genes.mit.edu/GENSCAN.html

8 http://gydb.org/index.php/Main_Page

9 http://www.ebi.ac.uk/clustalw

10 http://www.megasoftware.net

11 http://soybase.org/soytedb
